# Detection of *Anaplasma phagocytophilum* DNA in *Ixodes* Ticks (Acari: *Ixodidae*) from Madeira Island and Setúbal District, Mainland Portugal

**DOI:** 10.3201/eid1009.040276

**Published:** 2004-09

**Authors:** Ana Sofia Santos, Maria Margarida Santos-Silva, Victor Carlos Almeida, Fátima Bacellar, John Stephen Dumler

**Affiliations:** * Instituto Nacional de Saúde Dr. Ricardo Jorge, Águas de Moura, Portugal;; † Direcção Regional de Pecuária, Funchal, Portugal;; ‡ Johns Hopkins University School of Medicine, Baltimore, Maryland, USA

**Keywords:** Portugal, *Anaplasma phagocytophilum*, Polymerase chain reaction (PCR), Ticks, *Ixodes ricinus*, *Ixodes ventalloi*, research

## Abstract

A total of 278 *Ixodes* ticks, collected from Madeira Island and Setúbal District, mainland Portugal, were examined by polymerase chain reaction (PCR) for the presence of *Anaplasma phagocytophilum*. Six (4%) of 142 *Ixodes ricinus* nymphs collected in Madeira Island and 1 nymph and 1 male (2%) of 93 *I. ventalloi* collected in Setúbal District tested positive for *A. phagocytophilum msp2* genes or *rrs*. Infection was not detected among 43 *I. ricinus* on mainland Portugal. All PCR products were confirmed by nucleotide sequencing to be identical or to be most closely related to *A. phagocytophilum*. To our knowledge, this is the first evidence of *A. phagocytophilum* in ticks from Setúbal District, mainland Portugal, and the first documentation of *Anaplasma* infection in *I. ventalloi*. Moreover, these findings confirm the persistence of *A. phagocytophilum* in Madeira Island's *I. ricinus*.

## Abstract 2

*Anaplasma phagocytophilum* DNA is detected in Portuguese *Ixodes ricinus* and *I. ventalloi* ticks.

*Anaplasma phagocytophilum* (formerly *Ehrlichia phagocytophila*, *E. equi*, and the human granulocytic ehrlichiosis agent [HGE agent] [*1*]) is well established as a worldwide tickborne agent of veterinary importance and is considered an emerging human pathogen. The initial reports of human disease caused by *A. phagocytophilum*, now called human granulocytic anaplasmosis, came from Minnesota and Wisconsin in 1994 (*2**,**3*). Human granulocytic anaplasmosis is an acute, nonspecific febrile illness characterized by headache, myalgias, malaise, and hematologic abnormalities, such as thrombocytopenia and leukopenia as well as elevated levels of hepatic transaminases (*4*). Since that first report, an increasing number of cases have been described, mostly in the upper Midwest and in the Northeast regions of the United States (*5*). Three years later, in 1997, acute cases of this disease were also described in Europe (*6**,**7*). Several serologic and polymerase chain reaction (PCR)-based studies described the wide distribution of *A. phagocytophilum* across Europe and in some parts of the Middle East and Asia (*8**–**10*). Nevertheless, confirmed cases of human granulocytic anaplasmosis are rare; most European cases are described in Slovenia (*11*), with only a few reports from other European countries (*12*) and China (*13*).

The ecology of *A. phagocytophilum* is still being defined, but the agent is thought to be maintained in nature in a tick-rodent cycle, similar to that of *Borrelia burdgdorferi* (the agent of Lyme disease), with humans being involved only as incidental "dead-end" hosts (*14**–**17*). Exposure to tick bites is considered to be the most common route of human infection, although human granulocytic anaplasmosis has been reported after perinatal transmission or contact with infected animal blood (*18**,**19*). *A. phagocytophilum* is associated with *Ixodes* ticks that are known vectors, including *I. scapularis*, *I. pacificus*, and *I. spinipalpis* in the United States (*15**,**20**,**21*), *I. ricinus* mostly in southern, central and northern European regions (*22**–**26*), *I. trianguliceps* in the United Kingdom (*27*), and *Ixodes persulcatus* in eastern parts of Europe (*28*) and Asia (*9*).

In Portugal little information is available concerning the epidemiology of *A. phagocytophilum*; the agent was documented only once in *I. ricinus* ticks from Madeira Island (Núncio MS, et al, unpub data). However, the true prevalence and public health impact of *A. phagocytophilum* is likely underestimated since little research has been conducted on this bacterium in Portugal. In fact, seasonal outbreaks of enzootic abortions and unspecific febrile illness (commonly named pasture fever) in domestic ruminants, which could be attributable to *A. phagocytophilum*, have been known to breeders and veterinarians across the country for years. Thus, to expand knowledge of *A. phagocytophilum* in Portugal, a detailed investigation was initiated. The preliminary results concerning agent distribution are presented here. The purpose of this study was to investigate both the persistence of *A. phagocytophilum* on Madeira Island, where it was initially described, and the presence of the agent in *Ixodes* ticks from mainland Portugal.

## Materials and Methods

### Tick Sampling

**Figure 1:**
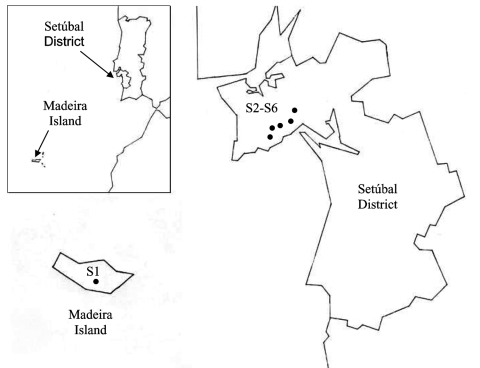
Collection sites in Madeira Island and Setúbal District, mainland Portugal. S, collection site.

During 2003 and the beginning of 2004, adults and nymphs were collected from one site on Madeira Island (site 1, Paúl da Serra–Porto Moniz) and from five different sites in the Setúbal District, mainland Portugal (site 2, Barris–Palmela; site 3, Baixa de Palmela; site 4, Picheleiros–Azeitão, site 5, Azeitão, site 6, Maçã–Sesimbra) (Figure 1). Most ticks were unfed, actively questing arthropods; they were obtained by flagging vegetation on pastures and wooded areas bordering farms and country houses. In site 3, additional specimens were also collected from domestic cats (*Felis catus domesticus*). The ticks were identified by morphologic characteristics according to standard taxonomic keys (*29**,**30*).

### Preparation of DNA Extracts from Ticks

Ticks were processed individually as described (*25*). Briefly, each tick was taken from the 70% ethanol solution used for storage, air dried, and boiled for 20 min in 100 µL of 0.7 mol/L ammonium hydroxide to free DNA. After cooling, the vial with the lysate was left open for 20 min at 90°C to evaporate the ammonia. The tick lysate was used directly for PCR. To monitor for occurrence of false-positive samples, negative controls were included during extraction of the tick DNA (one control sample for each six tick samples, with a minimum of two controls).

### PCR Amplification

DNA amplifications were performed in a Biometra T-3 thermoblock thermal cycler (Biometra GmbH, Göttingen, Germany) with two sets of primers: msp465f and msp980r, derived from the highly conserved regions of major surface protein-2 (*msp2*) paralogous genes of *A. phagocytophilum* (*31*), and ge9f and ge10r, which amplify a fragment of the 16S rRNA gene of *A. phagocytophilum* (*3*). PCR was performed in a total volume of 50 µL that contained 1 µmoL/L of each primer, 2.5 U of Taq DNA polymerase (Roche, Mannheim, Germany), 200 µmoL/L of each deoxynucleotide triphosphate (GeneAmp PCR Reagent Kit, Perkin-Elmer, Foster City, CA), 10 mmoL/L Tris HCL, 1.5 mmoL/L MgCl_2_, and 50 mmoL/L KCl pH 8.3 (Roche), as described (*3**,**31*). Adult ticks were tested individually by using 5 µL of DNA extract. Nymphs were pooled according to geographic site, up to a maximum of 10 different tick extracts per reaction, and 10 µL of the pooled DNA was used for initial screening. All positive pools were confirmed in a second PCR round that used 5 µL of original DNA extract from each nymph. PCR products were separated on 1.5% agarose by electrophorectic migration, stained with ethidium bromide, and visualized under UV light. Quality controls included both positive and negative controls that were PCR amplified in parallel with all specimens. To minimize contamination, DNA preparation with setup, PCR, and sample analysis were performed in three separate rooms.

### DNA Sequencing and Data Analysis

Each positive PCR product was sequenced after DNA purification by a MiniElute PCR Purification Kit (Qiagen, Valencia, CA). For DNA sequencing, the BigDye terminator cycle sequencing Ready Reaction Kit (Applied Biosystems, Foster City, CA), was used as recommended by the manufacturer. Sample amplifications were performed with the forward and reverse primers used for PCR identification (*3**,**31*), with the following modifications: 25 cycles of 96°C for 10 s, 4°C below the melting temperature of each primer for 5 s, and 60°C for 4 min. Dye Ex 96 Kit (Qiagen) was used to remove the dye terminators. Sequences were determined with a 3100 Genetic Analyzer sequencer (Applied Biosystems). After review and editing, sequence homology searches were made by BLASTN analysis of GenBank. Sequences were aligned by using ClustalX (*32*) with the neighbor-joining protocol and 1,000 bootstrap replications, and comparing with the 2 *msp2* paralogs of *A. phagocytophilum* Webster strain (AY253530 and AF443404), one *msp2* paralog of USG3 strain (AF029323), and with *A. marginale msp2* (AY138955) and *msp3* (AY127893) as outgroups. Dendrograms illustrating the similarity of *msp2*s were visualized with TreeView (*33*).

## Results

**Figure 2:**
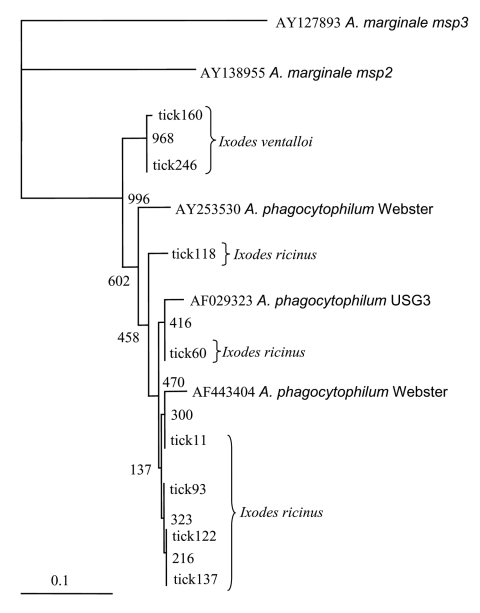
Dendrogram showing the phylogenetic relationships of the *msp2* sequences of the newly identified strains and other representative sequences from North American *Anaplasma phagocytophilum* strains (Webster strain–Wisconsin and USG3 strain–eastern United States), and from *A. marginale* Florida strain (*msp2* and *msp3*). Bootstrap values (out of 1,000 iterations) are shown at the nodes. Bar, substitutions/1,000 bp.

A total of 278 *Ixodes* ticks were tested for *A. phagocytophilum* DNA, including 142 *I. ricinus* from Madeira Island and 43 *I. ricinus* and 93 *I. ventalloi* from Setúbal District. The site of collection, origin, and tick stage are shown in Table 1 and Figure 1. PCR performed with the *msp2* primers detected *A. phagocytophilum* DNA in seven pools of nymphs (six pools of 10 *I. ricinus* from site 1, Madeira Island, and one pool of 4 *I. ventalloi* from site 3, Setúbal District) and also in 1 male *I. ventalloi* from site 3, Setúbal District, as demonstrated by the characteristic 550-bp band. PCRs conducted on individual ticks that comprised positive pools confirmed the results and showed that only one nymph per positive pool contained *A. phagocytophilum* DNA (Table 1 and Table 2). PCR test results were negative for all *I. ricinus* collected in the sites in Setúbal District. Overall, the infection rate was 6 (4%) of 142 for *I. ricinus* and 2 (2%) of 93 for *I. ventalloi*. Analysis based on direct amplicon sequencing showed the expected conserved 5´ end followed by ambiguous sequences that corresponded to the hypervariable central region of *msp2*, as anticipated based on the presence of >52 *msp2* copies in the *A. phagocytophilum* HZ strain genome (*34*). Thus, for appropriate comparison and alignment, the *msp2* 5´ sequences were edited from the positions where unambiguous reads could be determined and terminated 70 nt into the sequence at the approximate beginning of the hypervariable region. A similar alignment protocol for the 3´ end of the *msp2* amplicons showed more ambiguous positions, which prohibited effective alignment and sequence determination. Thus, *msp2* sequence alignments depended upon approximately 70 nt 5´> to the hypervariable region and were performed less for phylogenetic stratification of *A. phagocytophilum* in the ticks than to confirm that the amplified *msp2* sequences were not derived from other related *Anaplasma* or *Ehrlichia* spp. The nucleotide sequences determined for this 70-bp region amplified from all eight ticks showed 98.5%–85.7% similarity, 94.2%–86.9% similarity when compared to representative *msp2* sequences of *A. phagocytophilum* Webster and USG3 strains, and 63.7%–35.0% similarity when compared to *A. marginale msp2* and *msp3* sequences (Figure 2). Sequences obtained from the two *I. ventalloi* from mainland Portugal clustered together and separately from other *msp2* sequences obtained from *I. ricinus* on Madeira Island (Figure 2).

**Table 1:** Results of PCR to detect *Anaplasma phagocytophilum* DNA in ticks^a^

Area	Site	Origin	*Ixodes ricinus*			*I. ventalloi*			
			Nymphs^b^	F^b^	M^b^	Nymphs^b^	F^b^	M^b^	Total^c^
Madeira Island									
Paúl da Serra–Porto Moniz	1	Vegetation	6/139	0/2	0/1	–	–	–	142
Setúbal District Portugal Mainland									
Barris–Palmela	2	Vegetation	0/1	0/5	0/7	–	–	0/1	14
Baixa de Palmela	3	Vegetation	0/2	0/2	0/2	1/15	0/6	0/7	34
		*Felis catus domesticus*	–	–	–	–	0/6	1/4	10
Picheleiros–Azeitão	4	Vegetation	–	0/2	0/2	0/12	0/9	0/18	43
Azeitão	5	Vegetation	–	–	0/1	–	–	0/1	2
Maçã–Sesimbra	6	Vegetation	–	0/10	0/9	0/1	0/4	0/9	33
Total^c^			142	21	22	28	25	40	278

^a^PCR, polymerase chain reaction; F, female; M, male. ^b^Number of positives ticks/number of ticks examined. ^c^Total number of ticks examined.

**Table 2:** PCR-positive results of ticks^a^

Sites	No. positive nymphs	No. positive adults	Tick extracts codes
Madeira Island			
1	6	–	11; 60; 93; 118; 122; 137
Setúbal District Mainland Portugal			
3	1	1	160; 246 (respectively)

^a^PCR, polymerase chain reaction.

When amplified by using *rrs* primers ge9f and ge10r, compared to *A. phagocytophilum* U02521, sequences were 99% identical to two *I. ventalloi* (636/640 positions and 846/848 positions, respectively) on mainland Portugal and to three *I. ricinus* (836/841, 817/820, and 838/839 positions, respectively) on Madeira Island.

## Discussion

This study constitutes part of a larger effort to investigate the distribution of *A. phagocytophilum* in various regions of Portugal. Our data provide supporting evidence that *A. phagocytophilum* is present in actively questing *I. ricinus* from Madeira Island and in *I. ventalloi* from Setúbal District, mainland Portugal.

We used two approaches for identifying *A. phagocytophilum* in ticks: 1) standard amplification of *rrs* that can have limited sensitivity because of a single copy in each bacterial genome, and 2) amplification of *msp2*, a gene for which as many as 52 paralogs are present in the *A. phagocytophilum* genome and for which detection sensitivity is enhanced (*34*). The pitfall of *msp2* amplification derives from targeting conserved sequences that flank a hypervariable central region, which results in amplicons with partial sequence ambiguity when cloning is not attempted before sequencing (*31*). These findings are highly unlikely to represent amplicon contamination since marked sequence diversity was observed, and since only a single tick from each pool was positive in each reaction. Although only limited data can gleaned by this analysis, which interrogates only nucleic acids of small size, Casey et al. have shown that *msp2* "similarity" groups, reflecting clusters determined by a similar sequencing approach, can be useful in predicting phylogenetic relationships, particularly with reference to adaptation to specific host niches (*35*).

Madeira, the main island of the Madeira Archipelago, is located in the North Atlantic Ocean, about 800 km west of the African continent and 1,000 km from the European coast. On this island, *I. ricinus* is the most abundant tick species and the only *Ixodes* tick that was found in this study. *A. phagocytophilum* was detected in 4% of *I. ricinus* collected in Paúl da Serra. Our results corroborate previous findings, although prevalence here is slightly lower than the 7.5% infection rate in ticks previously collected in similar areas (Núncio MS, et al., unpub data). These differences may be attributable to seasonal variations in *A. phagocytophilum* prevalence within reservoir hosts or ticks or to technical aspects of detection. Regardless, studies that use a greater number of samples and that are performed in different seasons, locations, and habitats will be needed to confirm the levels of infection. Nevertheless, these findings are generally similar to those described elsewhere in Europe, although prevalence rates can vary greatly with the origin of *I. ricinus* examined, ranging from a minimum of <1% in the United Kingdom, France, and Sweden (*23**,**24**,**36*) to a maximum of 24% to 29% in northern Italy, Germany, and Spain (*22**,**25**,**26*). The public health importance of these findings still remains to be determined. *I. ricinus* is an exophilic, three-host tick known to bite several domestic animals and humans in Portugal (*30*). Therefore, we can assume that the presence of *A. phagocytophilum* on Madeira Island *I. ricinus* suggests a potential health threat to animals and humans and should be investigated.

Mainland Portugal is the most western region of Europe, with an area of 89,000 km^2^, divided into 18 districts. Although *I. ricinus* is not the main tick species in mainland Portugal, it can be found across the country in habitats with favorable conditions. Focused in Setúbal District, to the south of the Tejo River, our study detected *I. ricinus* in all five sites chosen for field work: Barris; Baixa de Palmela; Picheleiros; Azeitão, and Maçã. In those sites, the distribution of *I. ricinus* was accompanied by another *Ixodes* species, *I. ventalloi*. Another ecologically interesting finding that should be further confirmed was that, although all of the *I. ricinus* from mainland Portugal tested negative, evidence of *A. phagocytophilum* was found in 2% of all *I. ventalloi*, including 5% collected in Baixa de Palmela. The *msp2* sequences identified in these two ticks were more closely related to each other than to any *msp2* sequence identified in ticks from Madeira Island. In contrast, *A. phagocytophilum msp2* diversity in *I. ricinus* from Madeira Island was broad and showed overlap with gene sequences identified in North American strains, as observed for some *A. phagocytophilum* strains in the United Kingdom (*35*).

To our knowledge, this identification of *A. phagocytophilum* in ticks is the first from mainland Portugal and the first documentation of *Anaplasma* infection in *I. ventalloi*. This species is an endophilic, three-host tick well adapted to a broad range of habitats that vary from open, dry forest in semidesert Mediterranean areas to the mild humid conditions in the southern part of the British Isles. In Portugal, *I. ventalloi* infest a variety of small rodents, carnivores, and lizards but have not been found to feed on humans (*30*). *A. phagocytophilum* has already been reported in other ticks, besides the known vector species (*37**–**41*). The presence in alternate ticks is attributable to the existence of secondary maintenance cycles, in which *A. phagocytophilum* circulates between relatively host-specific, usually nonhuman-biting ticks and their hosts (*38**,**39*). Those additional cycles would buffer the agent from local extinction and help reestablish the primary cycles (*38**,**39*). Although this hypothesis might explain our results, the competency of *I. ventalloi* to act as vector for *A. phagocytophilum* has yet to be demonstrated. Moreover, the different average prevalences observed in each location suggest that *A. phagocytophilum* is not widely spread in ticks and that some reservoir animals or hosts are needed for its maintenance. Trapping and animal surveillance are needed to provide more information that could help to explain the biological importance of those findings.
